# Tunisian parents’ expectations and approaches regarding sex education of their children according to their age: a cross-sectional study

**DOI:** 10.1192/j.eurpsy.2024.777

**Published:** 2024-08-27

**Authors:** S. Rouached, M. Rouached, M. Lagha, S. Boudriga, A. Boumnijel, A. Dakhli, K. Zakhama, I. Ben Romdhane, W. Homri, R. Labbane

**Affiliations:** ^1^department C; ^2^department A, Razi psychiatric hospital, manouba, Tunisia

## Abstract

**Introduction:**

Adolescents often lack vital information for making wise sexual and reproductive decisions, leading to risks like abuse, unwanted pregnancies, and infections. Comprehensive, early, and age-appropriate sex education is crucial. While parents should play a significant role, many underestimate their responsibility. The perception of sex education is changing, with younger parents being more open to participating in their children’s education.

**Objectives:**

This study compares the approaches of Tunisian parents with adolescent and pre-adolescent children towards sex education.

**Methods:**

This study used a cross-sectional design to collect data from Tunisian parents of children between the ages of 1 to 18 using an online survey. Two groups were recruited based on the age of their children, one group had parents of children younger than 10 years old, and the other had parents of adolescents. The survey included questions about the participants’ demographics, approach to sex education, reasons for their approach, and opinions on sex-related education in public schools. The survey was anonymous and confidential, and data were collected from January to March 2023 through various social media platforms.

**Results:**

This study surveyed 232 Tunisian parents with children between the ages of 1 and 18, divided into two groups based on the age of their children. The majority of participants were female (62.1%) and married (81.9%). The majority of participants in both groups agreed that sex education is important and indispensable, but only 54.7% of parents in the older children group responded positively to teaching sexual education as an independent subject. There was a significant difference between the two groups regarding their opinions about the appropriate age of sexual education for their children, and who they think should discuss sexual and reproductive health with young people. Most participants indicated that the human body and its development, sexual and reproductive health, prevention of sexually transmitted diseases and infections, contraception as well as puberty are the most important subjects to be addressed. Sexuality and sexual behaviors, the concepts of violence and safety, interpersonal relationships, consent, insults, harassment, and sexist cyberbullying were less frequently mentioned.

**Conclusions:**

In conclusion, this study highlights the importance of sex education in Tunisia. Parents in both groups support it but differ on timing and integration. Barriers like communication challenges and religious beliefs exist. These insights can guide tailored sex education programs for Tunisian parents, promoting youth sexual and reproductive health.

**Disclosure of Interest:**

None Declared

